# Evaluation of intensified behaviour change communication strategies in an artemisinin resistance setting

**DOI:** 10.1186/s12936-016-1276-8

**Published:** 2016-04-30

**Authors:** Sara E. Canavati, Celine Zegers de Beyl, Po Ly, Muhammad Shafique, Thavrin Boukheng, Chandary Rang, Maxine Anne Whittaker, Arantxa Roca-Feltrer, David Sintasath

**Affiliations:** Malaria Consortium Cambodia, Phnom Penh Office, House #91, St. 95, Boeung Trabek, Chamcar Morn, Phnom Penh, Cambodia; Department of Clinical Tropical Medicine, Faculty of Tropical Medicine, Mahidol University, 420/6 Ratchawithi Road, Ratchathewi, Bangkok, 10400 Thailand; Centre for Biomedical Research, Burnet Institute, Melbourne, Australia; Malaria Consortium International, Development House, 56-64 Leonard Street, London, EC2A 4LT UK; The National Center For Parasitology, Entomology and Malaria Control, Ministry of Health, Corner street 92, Trapaing Svay village, Sankat Phnom Penh Thmey, Khan Sensok, Phnom Penh, Cambodia; Malaria Consortium Asia, Faculty of Tropical Medicine, Mahidol University, 420/6 Rajavidhi Road, Bangkok, 10400 Thailand; The University of Queensland, School of Public Health, Herston, QLD 4006 Australia; College of Public Health, Medical and Veterinary Sciences, James Cook University, Townsville, QLD 4006 Australia; Division of Tropical Health and Medicine, James Cook University, Townsville, QLD 4006 Australia

**Keywords:** Behaviour change communication strategy, Interpersonal communication, Artemisinin resistance, Malaria elimination, Health-seeking behaviour, Cambodia

## Abstract

**Background:**

In Cambodia, behaviour change communication (BCC) represents an integral component of malaria efforts aimed at fighting artemisinin resistant parasites and achieving elimination. The multi-pronged BCC interventions include interpersonal communication through village health volunteers (VHVs) and village malaria workers (VMWs), broadcasting malaria prevention, diagnosis and treatment messages via TV, radio and mobile broadcasting units (MBUs), distributing information education and communication (IEC) materials and introducing mobile malaria workers (MMWs) in endemic villages.

**Methods:**

This was a cross sectional household survey using a stratified multi-stage cluster sampling approach, conducted in December 2012. A stratified multi-stage cluster sampling approach was used; 30 villages were selected (15 in each stratum) and a total of 774 households were interviewed. This survey aimed to assess the potential added effect of ‘intense’ BCC interventions in three Western provinces. Conducted 2 years after start of these efforts, ‘non-intense’ BCC (niBBC) interventions (e.g., radio or TV) were compared to “intense” BCC (iBBC) implemented through a set of interpersonal communication strategies such as VMWs, VHVs, mobile broadcasting units and listener viewer clubs.

**Results:**

In both groups, the knowledge of the mode of malaria transmission was high (96.9 vs 97.2 %; p = 0.83), as well as of fever as a symptom (91.5 vs 93.5 %; p = 0.38). Knowledge of local risk factors, such as staying in the forest (39.7 vs 30.7 %; p = 0.17) or the farm (7.1 vs 5.1 %; p = 0.40) was low in both groups. Few respondents in either group knew that they must get tested if they suspected malaria (0.3 vs 0.1; p = 0.69). However, iBBC increased the discussions about malaria in the family (51.7 vs 35.8 %; p = 0.002) and reported prompt access to treatment in case of fever (77.1 vs 59.4 %; p < 0.01).

**Conclusion:**

The use of iBCC supported positive improvements in both attitudes and behaviours among the population with regard to malaria compared to mass media (niBCC) only. The significantly increase in people seeking treatment for fever in iBCC villages supports Objective Five of the Strategic Plan in the Cambodia Malaria Elimination Action Framework (2016–2020). Therefore, this study provides evidence for the planning and implementation of future BCC interventions to achieve the elimination of artemisinin resistant *Plasmodium falciparum* malaria.

**Electronic supplementary material:**

The online version of this article (doi:10.1186/s12936-016-1276-8) contains supplementary material, which is available to authorized users.

## Background

### What is behaviour change communication?

Behaviour change communication (BCC) is widely recognized as one of the main health promotion strategies [1, 2]. It is an interactive process of working with individuals and communities to develop communication strategies to promote positive behaviours as well as create a supportive environment to enable them to adopt and sustain positive behaviours [3]. The primary objectives of BCC in public health programmes [1], are to modify lifestyles which risk individual well-being, and to achieve health-improvements and environmental change [4]. Amongst the available BCC strategies to induce voluntary behaviour change, without economic or legal intervention, there are currently two options: interpersonal and mass communication [4, 5]. The most commonly accepted method is interpersonal communication through face-to-face education, either in individual or small group sessions, group teaching, and other techniques designed to influence the behaviour of participants [5–7].

### Evaluations of behaviour change communication

Human behaviours with relation to malaria are dynamic, they change in response to new policies, interventions and messages [8, 9]. To promote new malaria interventions and to keep the audience engaged in the BCC messages delivered evaluation research is urgently needed [9, 10]. Most evaluations conducted on BCC interventions have been primarily focused on the areas of family planning and HIV [9, 11–16]. Periodic national cross-sectional household surveys can provide the much-needed data on determinants of malaria behaviours, track the impact of BCC efforts, solidify and inform the evidence base, and allow us to adapt efforts to respond to a shifting malaria setting [9]. Swaziland is an example on how yearly knowledge, attitudes and practices (KAP) studies are helping to track progress, monitor the contribution of different communication channels, and focus communication activities on the most at-risk groups [17].

### Artemisinin resistance in Cambodia

In Cambodia, the control and elimination of malaria has garnered increased attention since the detection of artemisinin resistance in Pailin Province on the Thai border, in 2009 [18]. Today, the recommended anti-malarial treatment still relies on artemisinin and its derivatives [19, 20], which according to recent evidence is not only losing its effectiveness but the efficacy of the partner drug is also failing [21–23]. This geographic area has historically been the source of resistance to other anti-malarial drugs and if artemisinin resistance were also to spread globally, it would be a major threat to malaria control and elimination efforts worldwide [24, 25].

Behavioural barriers to malaria control are well-documented [26]: inconsistent or non-use of mosquito nets; delays in seeking effective treatment; and the inappropriate communication when distributing intermittent preventive therapy [27–36]. Integrating BCC components to key malaria interventions can help individuals and communities overcome these barriers [8, 9]. Different factors have contributed to the emergence of drug resistance on the Thailand–Cambodia border such as natural selection, substandard drugs, artemisinin monotherapies, lack of compliance to treatment course, and poor health-seeking behaviours [37, 38]. It has been well documented that anti-malarial drug use in western Cambodia has been irregular in terms of prescription practices of providers from the formal and informal sectors, as well as patient adherence to national treatment guidelines [37].

Any delay in receiving diagnosis and treatment, whatsoever the cause (i.e., poor acceptability of services, mobility of infected persons, or inadequate surveillance and response to outbreaks) can contribute to the development and spread of drug resistant malaria parasites [39–41]. Therefore, it is crucial to understand people’s knowledge of, attitudes towards and practices in malaria prevention, diagnosis and health-seeking behaviours to develop tailored BCC strategies for different population subgroups to support their use of preventive and control measures and thereby contribute towards the interruption of the spread of resistant parasite.

### Knowledge, attitudes and practices of Cambodian households

Research and large scale evaluations show that the population residing in western Cambodia have a high level of knowledge of malaria and key aspects of its prevention but this does not always translate into preventive malaria practices [42]. For example, after intense and costly efforts to distribute insecticide-treated nets (ITN), only 40–60 % of the population reported sleeping under an ITN in areas with evidence of delayed response to artemisinin-based therapies or at risk of the spread of resistant parasite [42]. This gap between high levels of knowledge but lower levels of practice is often referred as the knowledge-practices (KP) gap [43]. Providing the right information to the population is rarely sufficient to bring about a sustained change in behaviour [44–46] and impacting on malaria transmission. However, the intensification of malaria control interventions aiming to contain the spread of resistant parasite also provides an enabling environment, with an increased availability of services and commodities in households such as insecticide treated bed nets. Therefore, the timing was right for the intensification of BCC efforts, to support the change in behaviour to achieve the elimination objective.

### The BCC strategy in the target areas

Well-conducted planning must inform BCC interventions so that messages are targeted to key audiences, activities are founded on behavioural theories and formative research [1, 3, 9, 46]. As countries progress towards eliminating malaria, BCC strategies will need to be updated and adapted as transmission dynamics change and perception of risk is reduced [9]. The National Strategic Plan (NSP) for malaria elimination in Cambodia, defines the objective of the BCC strategy as “to ensure universal community awareness and behaviour change among the population at risk and support the containment of artemisinin resistant parasites and eliminate all forms of malaria through comprehensive BCC, community mobilization, and advocacy”. A range of BCC activities has been implemented by the National Programme from 2009 to the present. The BCC strategy represented an integral component of the containment project aiming at increasing awareness and improve health-seeking behaviours of communities to fight artemisinin resistant parasite [47].

**Fig. 1:**
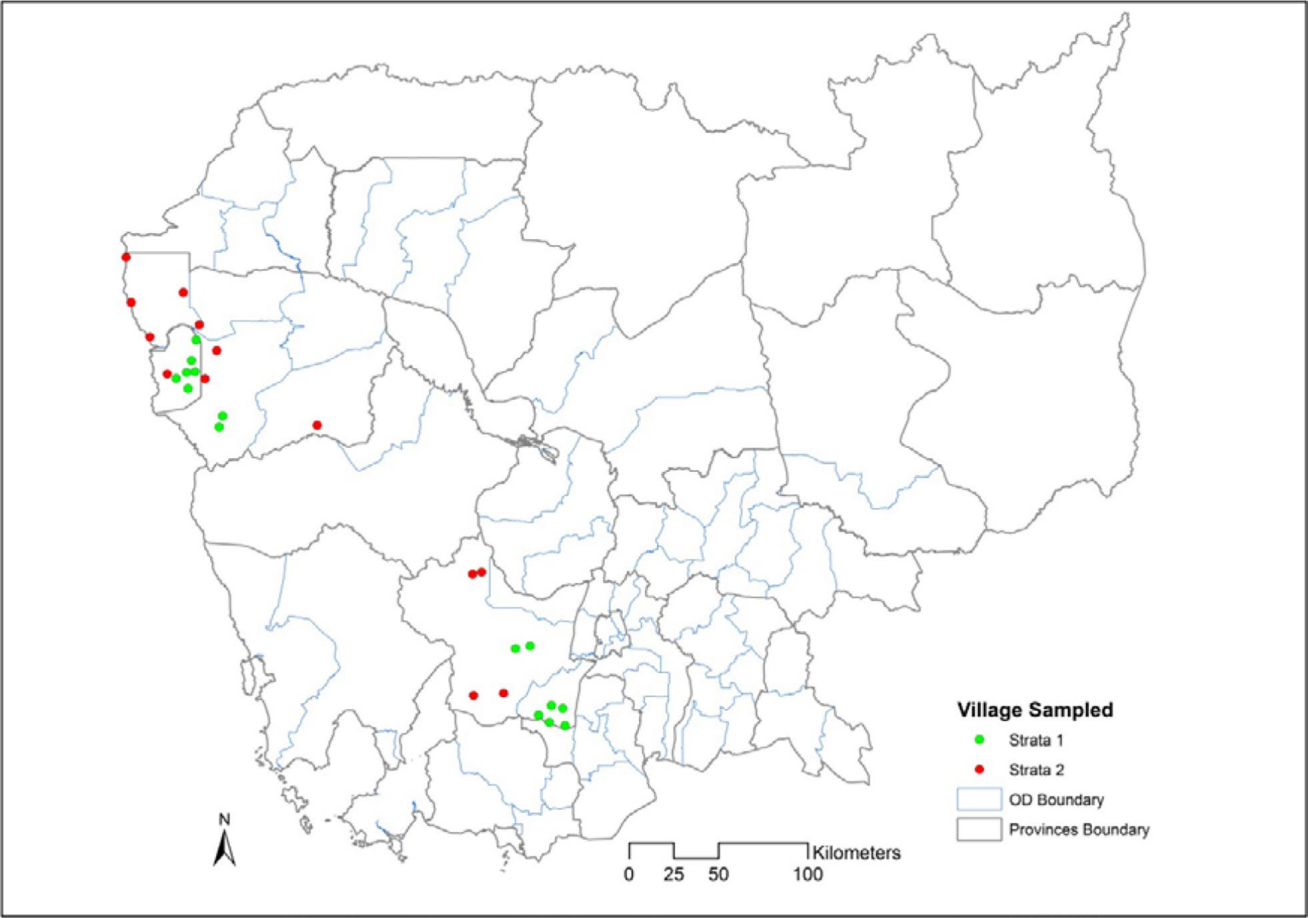
Location of the sampled iBCC and niBCC villages

In Cambodia, village malaria workers (VMWs) were first introduced in June 2001 as part of an ITN trial conducted by the National Centre for Parasitology, Entomology and Malaria Control (CNM) in Ratanakiri Province [48]. Between 2004 and 2005, the VMW scheme was rolled-out to cover 300 villages. Since then, three different approaches were tested and although a detailed comparison of feasibility and efficiency within the three intervention groups was never fully assessed. Findings indicated that it was feasible for these ‘expanded VMWs’ (eVMWs) to treat all three illnesses given proper training. Small, established rural communities in Cambodia also have a strong tradition of community participation [49]. This likely contributed to the reported success of the VMW project in Cambodia. The VMW project was launched in 2004 as a vertical national programme-led scheme that allowed essential supplies to bypass the delays and other problems inherent in the health system (Fig. 1).

With rising concern about the spread of drug resistance, a strategy of ‘intense-BCC’ activities was implemented by different organisations working in areas identified for “malaria containment”. These activities included new BCC messages about prevention, treatment and diagnosis, and correct malaria drug use via TV, radio and mobile broadcasting units (MBU), as well as the distribution of IEC material and a new focus on interpersonal communication (IPC) provided through a new cadre of community based volunteer—mobile malaria workers (MMW) in selected villages distributed along three provinces (Battambang, Kampong Speu and Pailin) [50]. The MMWs, introduced in 2009 building upon the VMW experiences in Cambodia are the backbone of all malaria interventions among MMPs.

### Purpose of this study

The aim of this assessment was to evaluate the outcomes and health promotion impact of BCC interventions after the first 2 years of implementation (2010–2012). The study objective was to assess knowledge, attitudes and practices of the population at-risk for malaria, between villages receiving ‘intense’ BCC interventions (iBCC) compared to villages receiving ‘non-intense’ BCC (niBCC) interventions. The hypothesis was that people living in villages targeted with iBCC interventions had higher knowledge levels and more likely to adopt the desired attitudes and practices towards malaria prevention and treatment compared to people living in villages only receiving niBCC messages.

## Methods

### Description of the intervention

“Intense” BCC intervention villages refer in this context to those villages that received direct community based BCC interventions in the form of interpersonal communication through VHVs, Mobile Broadcasting Units and listener viewer clubs (Table 1). “Non intense” BCC intervention villages include those villages which did not benefit from community based BCC activities but perhaps radio and/or television. These villages were used as comparison group to assess the impact of “intense” BCC on people behaviour. In intense villages, each VMW/MMW was monitored more closely, encouraged to do more active outreached and had more frequent training sessions.

**Table 1:** Description of activities in iBCC intervention

Intervention	Frequency	Duration	Messages	Delivery means
Community communication campaign	Two times per month	3–4 h per session	Malaria caused by mosquito bites Wear long cloth from dusk to dawn Sleep under ITN every night even you visit forest Always check blood test before treatment Say no to fake drug, it can kill	Competition Game, quiz Community rally Banners Music Storytelling Role play
Community media	Two times per month	1–2 h per session	Cause of malaria Prevention of malaria Proper treatment Use of treated bed nets Care of patient with fever	Video spot Role plays Game, quiz Song CD/DVD
Mobile broadcasting units (MBU)	Once a month (except for October due to heavy rain)	2 ½ h per session	Malaria prevention Malaria treatment Malaria diagnosis	MBU staff use video equipment to screen movies (drama/novels)
Listener viewer clubs	Once or twice a month	1 h per session	Malaria prevention Malaria treatment Malaria diagnosis	LVC staff use video equipment to screen movies (drama/novels)
IEC material: leaflets and banners	Once or twice a month	–	Malaria prevention Malaria treatment Malaria diagnosis	The leaflets are distributed during the LVC sessions and the banners will be located at the LVC post
Community focus group discussions (FGD)-education	Twice a month	½–1 h in each village	Cause of malaria Prevention of malaria Malaria caused by mosquito bites Wear long cloth from dusk to dawn Sleep under ITN every night even you visit forest Always check blood test before treatment	Big groups Small groups Door to door Face to face Poster Leaflets Flipchart
Village malaria BCC meeting with village health volunteers (VHV)	Once a month	1–2 h per session	Discussion on implementation issue and challenges	Groups discussion, report formats, practices on doing reports and field trip
Organize community meeting	Once a month	1–2 h per session	Sharing on implementation by village Refresh on lesion How to transfer the knowledge to target groups	Meeting, sharing experiences, knowledge refresher

Each non-governmental organization (NGO) implemented activities in line with its expertise as follows (Additional file 1):

(i) NGO 1 and 2 disseminated media products for broadcasting on radio and television and for community use targeting segmented population. One of these NGOs’ media products were broadcasted in target provinces as well as through Mobile Broadcasting Units and listener and viewer clubs (LVCs) in 30 target villages in two provinces (Pailin and Battambang).

(ii) NGO 3 worked with village health volunteers in Kampong Speu province to encourage preventive and positive health seeking behaviour in communities, build the capacity of health centre staff and improve the utilisation of the public health system for malaria diagnosis and treatment.

(iii) NGO 4 worked with VMWs and MMWs with a focus on continuing containment strategies in Pailin province as well as encouraging preventive and positive health-seeking behaviour in communities.

### Study design

This was a cross sectional household survey using a stratified multi-stage cluster sampling approach, where a “cluster” was defined as a “village”. The strata were defined according to “intense” or “non-intense” BCC interventions provided in the cluster. Each stratum was considered a survey domain from which 15 clusters were sampled, using probability proportionate to size method. In the second stage, household sampling was conducted using simple random sampling. All households were eligible for selection; a village list of households was used to randomly select an equal number of households in each cluster.

### Sample size

To calculate the sample size required, it was assumed that approximately 70 % of people living in the ‘intense’ targeted villages (intervention) would use an ITN (main outcome of interest was behaviour) compared to 50 % in the ‘non-intense’ targeted (control) villages. Assuming a precision of 0.05 and a design effect of two, a total of 178 households (HHs) was required in each stratum (N = 356). This meant that the sample size would, therefore, be sufficiently powered to detect a difference of at least 50–70 % between ‘intense’ (intervention) and ‘non-intense’ (control) BCC activities accordingly. However, in order to account for a potential 10 % refusal rate and to increase the power of the study to assess potential impact of single indicators between strata, the total sample size was increased to 390 households per cluster.

### Data collection

Interviewers were carefully selected so that they were culturally and socially acceptable. They had good working knowledge of Khmer language and previous experience in conducting household surveys. A 1-week training workshop including pilot-interviews was held prior to the field work. A detailed interview guide with the standard operating procedures was prepared to support the field team while conducting the interviews. The guide provided key ideas to interviewers on the conduct of interview and procedures to be followed by each team member.

Local authorities and community leaders were informed of the study once their cluster had been sampled. This included explanations about the purpose and expected time of the survey.

The female head of household was designated the primary respondent for this study. If no female respondent was available at the household after the third visit, a male respondent (>18 years old) was interviewed which happened much more in non-intense villages.

In total six teams were deployed for this assessment. Each team was allocated to one province and consisted of four people (one supervisor and three interviewers) accompanied with local workers (the village health worker and local authority) to assist in identifying the families to be interviewed. Each team had a supervisor who reported daily to the assessment coordinator.

### Data management and analysis

All information collected was double entered using a Microsoft Access 2010^®^ database purposively developed for this study. Both datasets were compared and any discrepant record was verified from the original questionnaires. Once this first stage of cleaning was finished, the data set was transferred to Stata version 10.1 (StataCorp LP, College Station, TX, USA) for further consistency checks and preparation for analysis. Final analysis consisted of basic frequencies and simple proportions and the McNemar test for significance was calculated, comparing the outcome indicators between sampling strata. All analysis accounted for sampling weights and any potential clustering effect using the “svyset” survey family command in Stata. In this paper, differences with a p value less than 0.05 will only be referred as significant.

### Ethical considerations

This assessment received ethical approval from the Cambodian National Ethics Committee for Health Research (NEHCR) in October 2012. Prior to each interview, the interviewer read the information sheet and consent form. Written informed consent was taken. This consent form contained information on the objectives of the survey, the risks, benefits and freedom of the participation, as well as information on confidentiality plus interviewee rights.

## Results

### Description of the sample

Household respondents were similar across stratum in terms of average age (40.9 vs 40.3 years), radio ownership (31.2 vs 30.3 %), marital status with most of them being married (83.3 vs 84.5 %) and ethnicity with the vast majority of them being Khmer (98.6 vs 100 %). In both strata, more than half of households had any member that travelled away from home and stayed overnight within the past 6 months (53.2 vs 52.8 %), indicating that this population is highly mobile. However, the two groups differed for some demographic and key social indicators. In iBCC villages, respondents were more likely to be female (73.8 %) while in niBCC villages respondent’s gender was more balanced (59.9 % females vs 40.1 % males) and this difference was statistically significant (p < 0.003). In intense BCC villages, the ownership of television was higher (55.0 vs 40.5 %) and there were slightly more farmers (82.1 vs 78.6 %) and fewer agricultural labourers (2.2 vs 6.4 %) (Table 2). Also, the level of education was low, with more respondents who had never attended school in intense BCC villages (23.6 vs 19.4 %). It is also worth noting that the Province also significantly differed across strata (p < 0.0001), but this was expected considering the study design.

**Table 2:** Characteristics of the household respondents

Background characteristic	iBCC N = 387 n (%)	niBCC N = 387 n (%)	Total N = 774 n (%)	Mc Nemar p value
Average age in years mean (range)	40.9 (12–77)	40.3 (14–80)	40.3 (12–80)	0.05*
Sex				
Male	104 (26.2)	146 (40.1)	250 (39.0)	0.003**
Female	283 (73.8)	241 (59.9)	524 (61.0)	
Ethnic group				
Khmer	382 (98.6)	387 (100)	769 (99.9)	0.0001***
Cham	4 (1.1)	0 (–)	4 (0.1)	
Vietnamese	1 (0.3)	0 (–)	1 (0.0)	
Live in a house that owns				
a radio	122 (31.2)	118 (30.3)	240 (30.3)	0.87
a television	209 (55.0)	152 (40.5)	361 (41.6)	0.10
Province				
Battambang	52 (4.8)	232 (79.1)	284 (73.3)	0.0001***
Pailin	156 (40.8)	26 (1.9)	182 (5.0)	
Kampong Speu	179 (54.3)	129 (19.0)	308 (21.8)	
Anyone from household travelled in past 6 months	209 (53.9)	206 (52.8)	415 (52.9)	0.80
Marital status				
Single-never married	14 (3.9)	18 (5.4)	32 (5.3)	0.11
Married	312 (83.3)	333 (84.5)	654 (84.4)	
Widowed	44 (10.8)	35 (9.8)	79 (9.9)	
Divorced/separated	7 (1.9)	1 (0.3)	8 (0.5)	
Married but not living together	1 (0.1)	0 (–)	1 (0.0)	
Occupation				
Agricultural labourer	8 (2.2)	20 (6.4)	28 (6.1)	0.73
Seller	26 (7.3)	23 (6.3)	49 (6.4)	
Fisherman	0 (–)	1 (0.1)	1 (0.1)	
Forestry worker (logging)	0 (–)	3 (1.0)	3 (0.9)	
Farmer	324 (82.1)	311 (78.6)	635 (78.9)	
Housewife	12 (3.3)	15 (4.1)	27 (4.0)	
Government staff	8 (2.4)	5 (1.2)	13 (1.3)	
Other	9 (2.6)	9 (2.2)	18 (2.3)	
Level of education				
Never attended school	92 (23.6)	85 (19.4)	177 (19.7)	0.19
1–3 years	99 (25.4)	105 (28.4)	204 (28.2)	
3 years or more	192 (49.8)	194 (51.4)	386 (51.3)	
University level	3 (0.9)	3 (0.8)	6 (0.8)	
Don’t know	1 (0.3)	0 (–)	1 (0.0)	

*Significant at the 0.05 probability level

**Significant at the 0.01 probability level

***Significant at the 0.001 probability level

### Level of knowledge

Overall, the knowledge of malaria signs was high with more than 85 % in each stratum citing both fever and chill (Table 3). More than 95 % of respondents in both groups knew that malaria was transmitted by mosquito. The levels of understanding of risk factors such as staying in the forest or on the farm was lower in general but tended to be higher amongst intense BCC villages than non-intense villages (39.7 vs 30.7 %) and (7.1 vs 5.1 %) respectively. More than 80 % of respondents in both areas knew that using an ITN can prevent malaria. In the intense BCC area, people were more likely to answer that the avoidance of mosquitoes (69.3 vs 58.4 %) and staying out of the forest (17.3 vs 12.7 %) are effective measures to avoid malaria.

**Table 3:** Comparison of household respondent’s knowledge of malaria

Indicator	iBCC N = 387		niBCC N = 387		Mc Nemar p value
	%	95 % CI	%	95 % CI	
Sign knowledge					
Fever	91.5	87.4–94.4	93.5	89.8–96.0	0.38
Chill	89.7	85.4–92.8	89.3	83.9–93.1	0.90
Know fever + chill	85.8	80.7–89.7	87.5	82.1–91.5	0.59
Transmission knowledge					
Malaria transmitted by mosquito	96.9	94.4–98.4	97.2	94.6–98.6	0.83
Staying/sleeping in forest	39.7	29.5–50.8	30.7	23.7–38.7	0.17
Staying sleeping in farm	7.1	4.0–12.3	5.1	2.8–9.0	0.40
Prevention knowledge					
Avoid mosquito bite	69.3	61.4–76.2	58.4	48.1–68.1	0.08
Sleep under an ITN	80.4	73.6–85.8	84.8	80.1–88.6	0.23
Stay out of forest	17.3	12.3–23.6	12.7	8.9–17.7	0.19
Reaction in case of malaria suspicion					
Stay home and wait for fever to go away	34.9	19.1–54.9	34.8	21.0–51.7	0.99
Seek advice from VMW/VMW	62.9	52.8–72.1	65.2	53.1–75.6	0.76
Seek advice from public health facility	15.5	8.1–27.5	21.0	12.2–33.6	0.44
Get a test	0.3	0.0–2.0	0.1	0.0–1.2	0.69
Appropriate source for test and treatment^a^					
Appropriate source for test^a^	90.4	81.8–95.2	91.1	84.7–95.0	0.87
Appropriate source for treatment^a^	88.5	78.7–94.1	91.5	85.6–95.1	0.47
Appropriate source for test AND treatment^a^	88.0	78.1–93.7	90.3	84.1–94.2	0.59
Knowledge of treatment duration					
Less than 3 days	0.4	0.1–1.7	0.3	0.0–2.4	0.08
3 days	58.6	50.1–66.6	57.8	48.1–67.0	
More than 3 days	16.6	11.9–22.7	8.8	5.7–13.4	
Don’t know	24.4	16.2–35.1	33.1	25.6–41.6	
Ever heard of resistance of malaria drugs	63.9	54.8–72.1	54.6	46.7–62.2	0.11

^a^Appropriate source = VMW/VHV, health centre/FDH, referral hospital or farm owner

If malaria was suspected respondents typically described staying at home and waiting for the fever to go away, seeking advice from VMW and seeking advice from a public health facility. Fewer than 0.5 % of respondents in both groups stated that they would seek to confirm the diagnosis by a malaria test. Knowledge of appropriate source for test and treatment was over 85 % in each stratum. Although the level of knowledge of the correct treatment duration (3 days) was similar across group, respondents in intense BCC villages were more likely to incorrectly answer “more than 3 days” (16.6 vs 8.8 %) but fewer of them did not know about the treatment duration (24.4 vs 33.1 %). Lastly, there were a higher proportion of respondents who had ever heard of drug resistant malaria in intense BCC villages (63.9 vs 54.6 %). However, none of these differences was statistically significant at the 5 % level (Table 3).

### Home practices and behaviours

The survey sought information on actual preventive behaviours and their attitudes towards these (Table 4). More respondents in the intense BCC villages stated that they had previously discussed malaria with another person (this could include the VMW or MMW as well) (51.7 vs 35.8 %) and this difference was statistically significant (p = 0.02). Substantially more households in the niBCC villages stated that they owned at least one ITN (94.6 vs 55.8 %; p < 0.01). Among those households who reported owning one or more ITN, the majority of respondents stated that all people in their household had used an ITN the previous night. ITN use was slightly higher in the niBCC villages (90.9 vs 84.5 %). This relationship varied when asked about regularity of use in the past week, where more of households in intense BCC group reported frequent use of the ITN (93.0 vs 91.7 %). Neither of these relationships was statistically significant at the 5 % level.

**Table 4:** Comparison of household respondent’s attitudes and reported behaviours

Indicator	iBCC N = 387		niBCC N = 387		Mc Nemar p value
	%	95 % CI	%	95 % CI	
Ever discussed about malaria	51.7	42.5–60.9	35.8	27.7–44.9	0.02
Household with any insecticidal net	55.8	33.2–76.2	94.6	85.1–98.1	<0.01**
ITN use in household last night among ITN owning households	N = 208		N = 368		
Nobody used an ITN	5.9	2.4–13.8	3.4	1.2–9.5	0.33
Some people used an ITN	9.6	4.6–18.9	5.7	3.7–8.6	
All people used an ITN	84.5	70.2–92.7	90.9	85.7–94.4	
ITN use frequency the past week	N = 208		N = 368		
Did not use at all	1.9	0.6–5.4	5.2	2.5–10.4	0.21
1–3 nights	2.9	1.2–7.0	2.3	1.2–4.5	
4–6 nights	93.0	85.8–96.7	91.7	86.0–95.2	
Every night	2.2	0.8–6.0	0.8	0.2–2.7	

**Significant at the 0.01 probability level

### Health seeking behaviour of reported fever cases

There were higher levels of fever in the past 1 month reported from the households in iBCC villages (31.4 vs 26.0 %). The majority of these fever cases were reported to have sought advice or treatment and the proportion was similar across group (94.1 vs 93.8 %) (Table 5). However, more respondents in the iBCC villages reported that health care was sought promptly, within 24 h of the onset of symptoms (77.1 vs 59.4 %). However, no more than half of these households reported having had a test for malaria usually reported as a rapid diagnostic test (RDT) but diagnosis confirmation was more common in the niBCC villages (50.5 vs 40.8 %). The test positivity rate was 27.9 and 24.5 % in intense and niBCC villages, respectively.

**Table 5:** Advice or treatment seeking behaviour of reported fever cases in past month

Indicator	iBCC N = 387		niBCC N = 387		Mc Nemar p value
	%	95 % CI	%	95 % CI	
Household with any reported fever case in past month	31.4	23.7–40.4	26.0	19.0–34.6	0.34
Sought any advice or treatment	N = 123		N = 97		
	94.1	89.3–96.9	93.8	87.3–97.1	0.91
When sought advice or treatment	N = 115		N = 90		
Same day	32.4	22.7–43.9	13.4	8.1–21.3	<0.01**
1 day later	44.7	34.8–55.0	46.0	35.4–56.9	
2 days later	20.1	13.2–29.4	32.0	22.7– 3.1	
3 days or more later	2.8	0.6–11.7	8.6	5.3–13.7	
Sought treatment within 24 h	77.1	68.5–83.9	59.4	48.3–69.6	<0.01**
Had any test for malaria	N = 115		N = 90		
	40.8	26.9–56.4	50.5	37.9–63.1	0.33
Test type	N = 47		N = 43		
RDT/dipstick	54.0	37.0–70.1	48.2	34.7–61.9	
Blood slide/microscopy	34.4	16.9–57.6	38.0	23.5–54.9	0.88
Don’t know	11.5	4.9–24.8	13.8	3.8–39.3	
Test result	N = 47		N = 43		
Positive	27.9	12.8–50.3	24.5	8.4–53.5	0.82
Negative	72.1	49.7–87.2	75.5	46.5–91.6	

**Significant at the 0.01 probability level

## Discussion

This study shows that use of iBCC supported positive improvements in both attitudes and behaviours among the population with regard to malaria compared to mass media (niBCC) only.

### Impact of intense BCC on knowledge level

These results show an overall high level of general knowledge on transmission mode, signs and symptoms and the use of ITNs as prevention tool in the two groups. There was no difference between the groups. However, iBCC messaging proved effective at raising knowledge about more subtle messages such as local risk factors such as the forest or the farm, or the phenomenon of malaria drug resistance. On the other hand, knowledge on malaria diagnostic and case management was generally low and no difference was found between groups. These findings are consistent with other survey results in the same area. Indeed, an evaluation of positive deviance to prevent malaria in 2012 [51] revealed similar proportions of household respondents knowing malaria symptoms (96.2 vs 93.5 % in niBCC group), malaria transmission mode (98.1 vs 97.2 % in niBCC group) and forest as a risk factor (28.6 vs 30.7 % in niBCC group). This could suggest that the targeted communities have reached the saturation level in regards to “traditional” BCC messaging, delivered by malaria programmes in this area since 2009, with the start of the containment activities. It shows that raising the knowledge level of the population is possible, with sustained iBCC activities over time.

### Impact of intense BCC on home practice and health seeking behaviours

iBCC had a significant positive impact on the frequency of discussion about malaria within the family. It also slightly increased the frequency of ITN use in the past week. Most striking, people with reported fever cases (in the last 6 months) in iBCC villages were more likely to report having accessed health care promptly, or on the same day. The difference across group was substantial (77.1 in iBCC vs 59.4 % in niBCC group) and the results observed in the iBCC group followed the same trend to what was estimated in the evaluation of positive deviance conducted in 2012 [51] (77.1 vs 55.6 %). Indeed, it is essential that people experiencing a fever episode suspect a malaria infection and report to a health practitioner if the resistant parasite is to be contained and ultimately eliminated.

Reported ITN use (in the last 24 h) was slightly higher (which was not statistical significant) in the niBCC villages (90.9 vs 84.5 %). However, in iBCC group reported frequent use of the ITN. Also, there were some differences in access to ITNs between intervention and non-interventions villages. Indeed, some villages in Kampong Speu province (intense area) were missed out during the mass distribution campaign of 2012. This could most probably explain the lower ITN ownership in the iBCC villages (55.8 vs 94.6 %) and hence the lower reported use a net the previous night. This difference highlights the importance of ensuring that health promotional activities are also supported by programmatic activities so that people have access to the tools, such as ITNs, required to actually change their behaviour, particularly when they are encouraged to do so by the BCC messaging.

The level of knowledge of the correct treatment duration and respondents who had ever heard of drug resistant malaria were lower in iBCC villages. More men were the target of the intensive communications (i.e., farmers, plantation workers, seasonal workers) but women were interviewed as men were out in the fields.

Future evaluations need to carefully identify the target populations for the interventions, and carefully sample within those groups. There may be challenges due to mobility of individuals, and if the interventions “creep” into the control groups, which can occur with media linked activities [52]. Qualitative data tools to complement the quantitative surveys may assist in providing a more in-depth and nuanced version of the impact of these additional intensive BCC activities [53]. The subtle changes in adherence, peer support for changed behaviour, reinforcement of messages when people may start to revert to old behaviours may be difficult to capture with survey tools [9].

The design of BCC interventions which are trying to sustain and reinforce behaviours changes (behavioural endpoints), not focus on knowledge improvements to support adoption (knowledge endpoints), may need to be more targeted and segmented e.g., reinforce messages of using a bed net every day to the women who will prepare the beds for the family on a daily basis. This focused approach will then assist evaluations find the subtle differences being sought [9].

### Comparability

The findings need to be considered in light of some differences between the communities served by the iBCC and niBCC activities. The populations receiving the intense interventions were living in areas known as Tier 1, where artemisinin resistance has been confirmed [54]. Consequently, these villages have been prioritized by malaria control and containment efforts. It is therefore logical to think that these households may be more aware of and concerned about malaria and therefore be more receptive to malaria messages than the rest of the population. The increase in access to malaria interventions, such as MMW, to test suspected cases and treat malaria infections may account for some of the increase in reported promptness of seeking care in the intense villages, and not be solely or evenly predominantly a factor of the more iBCC interventions. However, considering that both areas where within Tier 1, internal differences between iBCC and niBCC are likely not to be influenced by this factor. In addition, even if some degree of confounding effect cannot be entirely ruled out; however, considering that both areas where within Tier 1, internal differences between iBCC and niBCC are likely not to be influenced by this factor. The consistency of the results in comparison with other survey results and the magnitude of the difference in prompt access to health care indicate that these promising findings can be at least in part attributed to iBCC activities.

Ownership of television was higher amongst iBCC area (55 vs 40.5 %) which could have influenced how much information was provided to the recipients and accounted to some extent for the positive results of access to health care. However, this is unlikely as there was no difference across groups for knowledge indicators observed. In addition, considering that the national programme has invested significant resources into developing mobile broadcasting units, especially to target ‘dark media’ zones, it is unlikely that the difference in television ownership influenced the results.

Lastly, in iBCC villages, the level of education was low perhaps because the majority of respondents were women and it is well known that women in Cambodia have lower educational achievements [55].

### Potential limitations of the survey design

Reported fever cases in iBCC villages were more likely to report having accessed health care promptly, or the same day. However, this question was asked in the last 6 months so recall bias is likely to have played a role here. Recall bias can be reduced through the use of interviewing techniques and the design of the study protocol, including the tools. Also the investigator can increase the motivation of respondents [56]. Considering that standard methods were used for this data collection, ensuring consistency of approaches across strata, it is logical to think that this phenomenon would affect equally both strata and to a limited scale that would not greatly alter the key finding. However, these specific questions in the data collection tool purposely specified a recall period of 6 months for BCC messaging and of 1 month for the health seeking practices of reported fever cases. These questions were consistent with other similar assessments and these recall periods are now considered appropriate for the collection of reliable data in this field of research [42, 57–59].

The sampling of villages was done using the probability proportionate to size methodology. The largest villages were in two provinces, Pailin and Kampong Speu, where coincidentally the iBCC interventions were also concentrated. This may have limited the representativeness of the samples.

Treatment-seeking behaviour of individuals was investigated at household level, interviewing one household respondent. The possibility of respondent bias resulting in a potential clustering of fever cases within households cannot be ruled out. Indeed, within the previous month, several people could have been ill and that fever episode among adult or older age groups been unnoticed or unreported by the respondent. However, this potential occurrence would equally affect both strata and, therefore, would not affect the results.

## Conclusion

This study demonstrated that iBCC intervention using a set of interpersonal communication strategies was more effective in promoting positive attitude and behaviour among the population in regard to malaria than relying on mass media only. The finding was valuable because it provides evidence of an improvement in behaviour endpoints with more intense and interpersonal BCC activities and not just on knowledge endpoints as usually reported in BCC studies. The findings from this assessment support Objective five of the Strategic Plan in the Cambodia Malaria Elimination Action Framework (2016–2020): “to implementing comprehensive IEC/BCC approach that facilitates at least 90 % of people seeking treatment for fever within 24 h at a health facility or with a qualified care provider and at least 85 % of at-risk population utilizing an appropriate protection tool by 2017” [60]. This could be useful to the national malaria programme with regard to evidence-based decision-making for the planning and implementation of future BCC interventions and better targeting of messages and audiences, and ultimately contribute to the elimination of resistant malaria in these areas and prevent further spread regionally. Elimination of drug resistant malaria, and sustaining behaviours that prevent infection and support early diagnosis and treatment are key strategies to achieve malaria elimination in Cambodia and the broader regional strategy to have an Asia–Pacific free of malaria by 2030.

## Additional file

10.1186/s12936-016-1276-8 BCC activities by province and sub-sub-recipient (SSR).

## Abbreviations

BCC: behaviour change communication
CNM: National Centre for Parasitology, Entomology and Malaria Control
eVMW: expanded village malaria worker
HH: household
iBCC: intense behaviour change communication
IEC: information, education and communication
IPC: interpersonal communication
ITN: insecticide-treated net
LVC: listener and viewer club
KAP: knowledge, attitudes and practices
KP: knowledge-practices
MBU: Mobile Broadcasting Unit
MMW: mobile malaria worker
NEHCR: National Ethics Committee for Health Research
NGO: non-governmental organization
niBCC: non-intense behaviour change communication
NSP: National Strategic Plan
VHV: village health volunteer
VMW: village malaria worker

## References

[1] McAlister AL (1991). Population behavior change: a theory-based approach. J Public Health Policy.

[2] Conner M, Norman P (1995). Predicting health behaviour: research and practice with social cognition models.

[3] Rimer BK, Glanz K, Lewis FM, Rimer BK (1990). Perspectives on intrapersonal theories in health education and health behavior. health behavior and health education: theory, research, and practice.

[4] Bracht N (1990). health promotion at the community level.

[5] Ajzen I, Fishbein M (1980). Understanding attitudes and predicting social behavior.

[6] Fishbein M, Ajzen I (1975). Belief, attitude, intention, and behavior.

[7] Ariey F, Witkowski B, Amaratunga C, Beghain J, Langlois AC, Khim N (2014). A molecular marker of artemisinin-resistant *Plasmodium falciparum* malaria. Nature.

[8] Shelton J (2013). The six domains of behavior change: the missing health system building block. Glob Health Sci Pract.

[9] Koenker H, Keating J, Alilio M, Acosta A, Lynch M, Nafo-Traore F (2014). Strategic roles for behaviour change communication in a changing malaria landscape. Malar J.

[10] Tacchi J, Lennie J, Wilkins KG, Tufte T, Obregon R (2014). A participatory framework for researching and evaluating communication for development and social change. The handbook of development communication and social change.

[11] Bertrand J, Kincaid D (1997). Evaluating information, education, and communication in family planning, the evaluation project.

[12] Westoff C, Bankole A (1997). Mass media and reproductive behavior in Africa, DHS analytical reports No 2.

[13] Rogers E, Vaughan P, Swalehe R, Rao N, Svenkerud P, Sood S (1999). Effects of an entertainment-education radio soap opera on family planning behavior in Tanzania. Stud Fam Plann.

[14] Bertrand J, O’Reilly K, Denison J, Anhang R, Sweat M (2006). Systematic review of the effectiveness of mass communication programs to change HIV/AIDS-related behaviors in developing countries. Health Educ Res.

[15] Wakefield M, Loken B, Hornik R (2010). Use of mass media campaigns to change health behaviour. Lancet.

[16] Snyder L (2007). Health communication campaigns and their impact on behavior. J Nutr Educ Behav.

[17] Roll Back Malaria (2012). Focus on Swaziland.

[18] Dondorp A, Nosten F, Poravuth Y, Das D, Phyo A, Tarning J (2009). Artemisinin resistance in *Plasmodium falciparum* malaria. N Engl J Med.

[19] White N (2008). Qinghaosu (artemisinin): the price of success. Science.

[20] Maude RJ, Woodrow CJ, White LJ (2010). Artemisinin antimalarials: preserving the “magic bullet”. Drug Dev Res.

[21] Leang R, Barrette A, Bouth DM, Menard D, Abdur R, Duong S (2013). Efficacy of dihydroartemisinin-piperaquine for treatment of uncomplicated *Plasmodium falciparum* and *Plasmodium vivax* in Cambodia, 2008–2010. Antimicrob Agents Chemother.

[22] Saunders DL, Vanachayangkul P, Lon C (2014). Dihydroartemisinin piperaquine failure in Cambodia. N Engl J Med.

[23] Spring MD, Lin JT, Manning JE, Vanachayangkul P, Somethy S, Bun R (2015). Dihydroartemisinin-piperaquine failure associated with a triple mutant including kelch13 C580Y in Cambodia: an observational cohort study. Lancet Infect Dis.

[24] White NJ (2010). Artemisinin resistance–the clock is ticking. Lancet.

[25] Dondorp AM, Fairhurst RM, Slutsker L, Macarthur JR, Breman JG, Guerin PJ (2011). The threat of artemisinin-resistant malaria. N Engl J Med.

[26] Mwenesi H (2005). Social science research in malaria prevention, management and control in the last two decades: an overview. Acta Trop.

[27] Korenromp E, Miller J, Cibulskis R, Kabir Cham M, Alnwick D, Dye C (2003). Monitoring mosquito net coverage for malaria control in Africa: possession vs. use by children under 5 years. Trop Med Int Health.

[28] Widmar M, Nagel C, Ho D, Benziger P, Hennig N (2009). Determining and addressing obstacles to the effective use of long-lasting insecticide-impregnated nets in rural Tanzania. Malar J.

[29] Eisele T, Keating J, Littrell M, Larsen D, Macintyre K (2009). Assessment of insecticide-treated bednet use among children and pregnant women across 15 countries using standardized national surveys. Am J Trop Med Hyg.

[30] Githinji S, Herbst S, Kistemann T, Noor A (2010). Mosquito nets in a rural area of Western Kenya: ownership, use and quality. Malar J.

[31] Ngondi J, Graves P, Gebre T, Mosher A, Shargie E, Emerson P (2011). Which nets are being used: factors associated with mosquito net use in Amhara, Aromia and Southern Nations, Nationalities and Peoples’ Regions of Ethiopia. Malar J.

[32] Littrell M, Gatakaa H, Evance I, Poyer S, Njogu J, Solomon T (2011). Monitoring fever treatment behaviour and equitable access to effective medicines in the context of initiatives to improve ACT access: baseline results and implications for programming in six African countries. Malar J.

[33] Mangham L, Cundill B, Achonduh O, Ambebila J, Lele A, Metoh T (2011). Malaria prevalence and treatment of febrile patients at health facilities and medicine retailers in Cameroon. Trop Med Int Health.

[34] West P, Protopopoff N, Rowland M, Kirby M, Oxborough R, Mosha F (2012). Evaluation of a national universal coverage campaign of long-lasting insecticidal nets in a rural district in north-west Tanzania. Malar J.

[35] Namusoke F, Ntale M, Wahlgren M, Kironde F, Mirembe F (2012). Validity of self-reported use of sulphadoxine–pyrimethamine intermittent presumptive treatment during pregnancy (IPTp): a cross-sectional study. Malar J.

[36] Baltzell K, Elfving K, Shakely D, Ali A, Msellem M, Gulati S (2013). Febrile illness management in children under 5 years of age: a qualitative pilot study on primary health care workers’ practices in Zanzibar. Malar J.

[37] Yeung S, Van Damme W, Socheat D, White NJ, Mills A (2008). Cost of increasing access to artemisinin combination therapy: the Cambodian experience. Malar J.

[38] Yeung S, Van Damme W, Socheat D, White NJ, Mills A (2008). Access to artemisinin combination therapy for malaria in remote areas of Cambodia. Malar J.

[39] Bhumiratana A, Intarapuk A, Sorosjinda-Nunthawarasilp P, Maneekan P, Koyadun S (2013). Border malaria associated with multidrug resistance on Thailand–Myanmar and Thailand–Cambodia borders: transmission dynamic, vulnerability, and surveillance. Biomed Res Int.

[40] Martens P, Hall L (2000). Malaria on the move: human population movement and malaria transmission. Emerg Infect Dis.

[41] Lynch C, Roper C (2011). The transit phase of migration: circulation of malaria and its multidrug-resistant forms in Africa. PLoS Med.

[42] CNM. Report of the Cambodia containment survey 2009 and 2010.

[43] Launiala A (2009). How much can a KAP survey tell us about people’s knowledge, attitudes and practices? Some observations from medical anthropology research on malaria in pregnancy in Malawi. Anthropol Matters.

[44] Panter-Bricka CCS, Lomasa H, Pinderc M, Lindsay SW (2006). Culturally compelling strategies for behaviour change: a social ecology model and case study in malaria prevention. Soc Sci Med.

[45] Berggren W, Wray J (2002). Positive deviant behaviour and nutrition education. Food and Nutr Bull.

[46] Fishbein M, Yzer M (2003). Using theory to design effective behaviour change interventions. Commun Theory.

[47] CNM (2012). National BCC strategy.

[48] Sochantha T, Hewitt S, Nguon C, Okell L, Alexander N, Yeung S (2006). Insecticide-treated bednets for the prevention of *Plasmodium falciparum* malaria in Cambodia: a cluster-randomized trial. Trop Med Int Health.

[49] Brown E, Montavy C, Rattana H, Bundet S (2002). Health beliefs and practices with regards to malaria in ethnic minority communities in north-east Cambodia.

[50] Malaria Consortium. Workshop to consolidate lessons learned on BCC and mobile/migrant populations in the strategy to contain artemisinin resistant malaria. Meeting Report. Santi Resort & Spa Luang Prabang, Lao PDR5—7 July 2011.

[51] Shafique M (2012). Positive deviance inquiry on mobile and migrant workers in containment project, Cambodia.

[52] Gryseels C, Peeters Grietens K, Dierickx S, Xuan XN, Uk S, Bannister-Tyrrell M (2015). High mobility and low use of malaria preventive measures among the Jarai male youth along the Cambodia–Vietnam border. Am J Trop Med Hyg.

[53] Gryseels C, Uk S, Erhart A, Gerrets R, Sluydts V, Durnez L (2013). Injections, cocktails and diviners: therapeutic flexibility in the context of malaria elimination and drug resistance in Northeast Cambodia. PLoS One.

[54] Emergency response to artemisinin resistance in the Greater Mekong subregion. [http://www.who.int/malaria/areas/greater_mekong/en/].

[55] National Institute of Statistics DGfH, Macro I (2011). Cambodia Demographic and Health Survey 2010.

[56] Coughlin SS (1990). Recall bias in epidemiologic studies. J Clin Epidemiol.

[57] Dysoley L, Rithea L, Bunkea T, Babu S, Sim K, Nguon C, Survey Cambodia Malaria (2010). Phnom Penh.

[58] Thailand Malaria Survey 2012. Thailand: MC and BVDB; 2013.

[59] ACT Watch Group and PSI/Cambodia (2011). Kingdom of Cambodia household survey report, 2011.

[60] WHO (2016). Cambodia malaria elimination action framework (2016–2020).

