# Supplementary material for: Evaluation of intensified behaviour change communication strategies in an artemisinin resistance setting
Source: Malar J. 2016 Apr 30;15:249. doi: 10.1186/s12936-016-1276-8 (PMC4851777; doi:10.1186/s12936-016-1276-8)
Supplement: Supplementary file 1 — 10.1186/s12936-016-1276-8 BCC activities by province and sub-sub-recipient (SSR). [file 12936_2016_1276_MOESM1_ESM.docx]

*Supplementary Information*

**Supplementary Table 1. BCC activities by province and Sub-sub-recipient (SSR)**

| BCC Activities | Battambang | | | | Kampong Speu | | | | Pailin | | | |
| --- | --- | --- | --- | --- | --- | --- | --- | --- | --- | --- | --- | --- |
|  | NGO1 | NGO2 | NGO3 | NGO4 | NGO1 | NGO2 | NGO3 | NGO4 | NGO1 | NGO2 | NGO3 | NGO4 |
| Media Products for Broadcast on radio and television |  | X |  | X |  | X |  |  |  | X |  | X |
| IEC materials including, but not limited to, advertising through taxi stickers, flip charts, pamphlets, banners and billboards |  |  |  | X | X |  |  |  |  |  | X | X |
| Community campaigns |  |  |  |  | X |  |  |  |  |  | X |  |
| Mobile Broadcasting Units (MBUs) |  |  |  | X | X |  |  |  |  |  |  | X |
| Listener Viewer Clubs (LVCs) |  |  |  | X | X |  |  |  |  |  |  | X |
| Village Malaria Workers (VMWs) |  |  |  |  |  |  |  |  |  |  | X |  |
| Village Health Volunteers (VHVs) |  |  |  |  | X |  |  |  |  |  |  |  |
| Mobile Malaria Workers (MMWs) |  |  |  |  |  |  |  |  |  |  | X |  |
| Health System Strengthening (HSS) through health centre staff capacity |  |  |  |  | X |  |  |  |  |  |  |  |
| Improvement of the utilisation of the public health system for malaria diagnosis |  |  |  |  | X |  |  |  |  |  |  |  |
